# A protocol for analyzing repeated measures of online group behavior

**DOI:** 10.1016/j.mex.2022.101667

**Published:** 2022-03-22

**Authors:** Matthew G.R. Courtney, Jamie Costley, Mik Fanguy

**Affiliations:** a Graduate School of Education, Nazarbayev University, Nur-Sultan, Kazakhstan; b Institute of Education, National Research University Higher School of Economics, Moscow, Russia; c School of Humanities and Social Sciences, Korea Advanced Institute of Science and Technology (KAIST), Daejeon, South Korea

**Keywords:** Online collaborative group behavior, Repeated measures of nested data, Weeks in persons, Persons in groups, Two- and three-level multilevel models, R statistical programming, lme4, Lavaan

## Abstract

In this article, we feature a novel protocol that enables the analysis of repeated measures of online group behavior. The protocol accounts for (1) the nested hierarchy of the data with weeks nested in persons, and persons nested in weeks, and (2) the temporal nature of the behavior at the early, mid, and late periods of each week. To manage and analyze such data in a general way, we first give an illustration of the data structure. Thereafter, we propose a five-step Courtney-Fanguy-Costley protocol that (1) considers the data structure, (2) defines the levels of data, (3) considers variable variation, timing, and necessary aggregation, (4) ensures necessary variation, and (5) specifies null and mixed-effects models. We also provide exemplary R code for readers to replicate our approach.

• A general five-step protocol for analyzing repeated measures of online group behavior is offered.
• A description of the complex nested data structure is offered.
• Users can simulate data in R to run through the protocol.

## Graphical abstract

**Figure:**
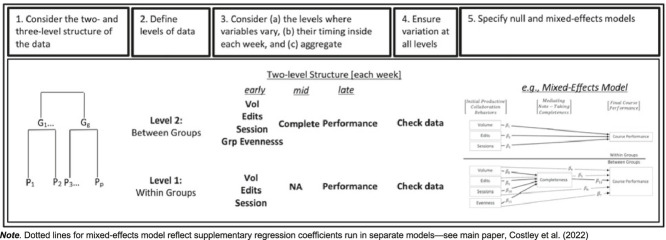

Specifications table

**Table:** 

Subject area	Psychology
More specific subject area	Instructional Design and Online Collaborative Learning
Method name	Courtney-Costley-Fanguy (CFC) Protocol for Analyzing Repeated Measures of Online Group Behavior
Name and reference of original method	Goldstein, H. (1987). *Multilevel models in educational and social research.* London: Griffin.
Resource availability	NA

## Method details

Educational research often asks, “What type of instructional design is most appropriate for enhancing learning?” With the onset of the Internet, student engagement with online platforms has become a topic of interest. Consequently, research questions vis-à-vis the most appropriate ways to group students and make use of online learning platforms have become popular (e.g., [4]).

More recently, it has become common for groups of students to make use of the openly available online platforms, such as Google Docs. Using this platform, course instructors can ask students to read, listen to, and watch instructional content; thereafter, working collaboratively in their small groups, classmates can be asked to take notes that capture and distill course content in a comprehensive way.

Various forms of student activity on the Google Docs platform, such as volume of words, number of edits, session logins, and group volume evenness can be tracked and archived on a weekly basis via the open-source software Google Docs add-on, DocuViz [2], and open-source Python code [3]. In addition, group performance, such as the completeness of the aforementioned group notes, can be assessed by way of validated writing rubrics [4]. Finally, individual students can also be examined by way of end-of-week quizzes (formal assessments) to provide a measure of student competence each week.

Multi-level modeling (Goldstein, [5]) provides a useful framework for analyzing data derived from such projects. This is because the data is complex and involves several nested hierarchies. For example, weekly variation in each student's volume of words contributes to an average number of words for each student. In addition, individual variation in the average number of words for each student contributes to an average number of words for that student's group.

For the research that the current paper is based on [4], student behavior was measured each week (a within-person variance component). However, further complicating the research designs was the fact that student behavior was also measured at the early, middle, and late stages of each week. Accounting for this longitudinal feature of the data is also important as we may be able to identify causal relationships that might be occurring each week. Therefore, prior to undertaking analysis, we also need to classify variables temporally.

Taken together, our Courtney-Fanguy-Costley (CFC) protocol calls for an understanding of the extent to which the full dataset varies (1) between-groups, between-persons, and within-persons, and (2) for each week, between-groups and within-groups. Table 1 provides an example of what this data structure looks like for the full dataset, and for the example subset single week dataset.

**Table 1:** Three- and two-level data structures for repeated measures of online group behavior.

Week	Person_ID	Group	Volume	Edits	Sessions	Grp_Evenness_PW	Complete	Perform
**Three-Level Structure [full dataset, all weeks]**								
1	1	1	**300**	**20**	**4**	**-9.21**	**75**	**8**
2	1	1	*450*	*40*	*5*	*-7.82*	*55*	*7*
3	1	1	*525*	*60*	*3*	*-8.63*	*50*	*6*
1	2	1	**400**	**30**	**6**	**-9.21**	**75**	**7**
2	2	1	*500*	*50*	*4*	*-7.82*	*55*	*6*
3	2	1	*600*	*70*	*5*	*-8.63*	*50*	*5*
1	3	1	**500**	**40**	**3**	**-9.21**	**75**	**6**
2	3	1	*550*	*60*	*2*	*-7.82*	*55*	*5*
3	3	1	*675*	*80*	*5*	*-8.63*	*50*	*4*
1	4	2	350	25	7	-20.65	80	9
2	4	2	*475*	*45*	*5*	*-6.44*	*65*	*8*
3	4	2	590	65	4	-4.61	95	7
1	5	2	4500	35	3	-20.65	80	8
2	5	2	*500*	*55*	*4*	*-6.44*	*65*	*7*
3	5	2	600	75	3	-4.61	95	6
1	6	2	55000	45	4	-20.65	80	7
2	6	2	*525*	*65*	*6*	*-6.44*	*65*	*6*
3	6	2	610	85	1	-4.61	95	5
**Two-Level Structure [each week]**								
1	1	1	**300**	**20**	**4**	**-9.21**	**75**	**8**
1	2	1	**400**	**30**	**6**	**-9.21**	**75**	**7**
1	3	1	**500**	**40**	**3**	**-9.21**	**75**	**6**
1	4	2	350	25	7	-20.65	80	9
1	5	2	4500	35	3	-20.65	80	8
1	6	2	55000	45	4	-20.65	80	7

*Note.* Group 1 for Week 1 is **bold**; Group 1 for Week 2 is **bold and underlined**; Group 1 for Week 3 is *italicized*; Group 2 for Week 1 is underlined; Group 2 for Week 2 is *italicized and underlined*; Group 2 for Week 3 is regular font.

At this juncture, let us briefly describe the variables in the full dataset for all weeks. Volume represents the total number of words produced at the early part of each week. Edits reflects the number of edits of another person's words for the same period. Likewise, Sessions represents the number of times the student logged in to contribute writing for that period. The Grp_Evenness_PW variable represents the degree to which the students in a group contributed an equal number of words. This variable is generated for each week and is calculated as the log of the reciprocal of the variance in Volume for that group for that week. For Group 1 for Week 1, the following R code illustrates how this variable can be calculated:

G1_W1_evenness <- log(1/(var(c(300,400,500)))) print(G1_W1_evenness) # -9.21034

The lower part of Table 1 presents the subset two-level data structure. For the research exemplified, student outcomes were measured at the group and individual level. For example, for the two-level data structure, the completeness of the group notes for person IDs 1, 2, and 3 for the middle of Week 1 do not change (i.e., 75, 75, & 75) as each person is in the same group and contributed to the same artefact. However, Performance, reflecting how well students do in quizzes at the late part of the week, changes dependent upon how well they do individually.

**Fig. 1:**
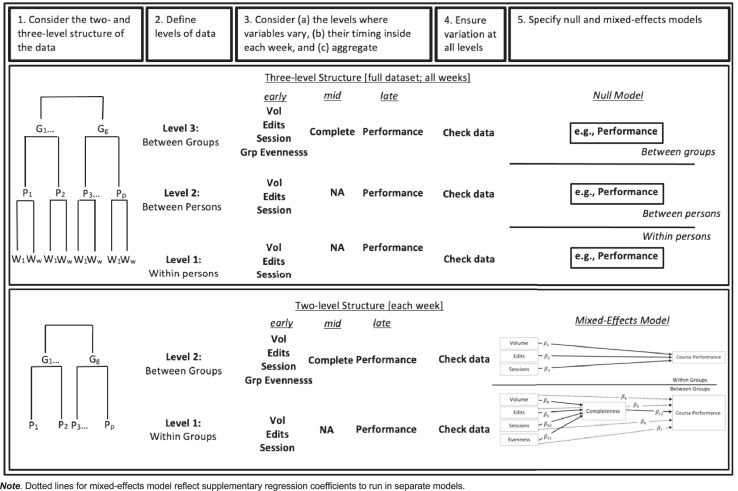
Courtney-Fanguy-Costley five-step protocol for analyzing repeated measures of online group behavior Note. Dotted lines for mixed-effects model reflect supplementary regression coefficients to run in separate models.

In this article, we contribute a general framework for researchers undertaking research projects with a similar design. Fig. 1 provides five steps that we adhere to in the approach coined the Courtney-Fanguy-Costley (CFC) protocol.

After gleaning data from Google Docs using DocuViz [2] and a customized set of procedures written in the Python programming language [1], application of the CFC protocol includes five steps.

In the first step, the two- and three-level structure of the data is sketched and considered. For the Costley et al. [4] study, the illustrated two- and three-level data structures were considered of research interest: The three-level structure allows for an overall comprehensive understanding of how student online behavior varied for the entire research project, while weekly two-level models provides a useful way to look at the temporal, potential causal, effects each week.

In the second step, each of the levels are appropriately defined. In the third step, we consider (a) the levels where variables vary, (b) their timing inside each week, and (c) aggregate where necessary. To illustrate (a), for the two-level structure, the Complete variable varies between groups but does not vary within groups (NA). To illustrate (b), for the same structure, student Vol (Volume) is early in the week, Complete(ness) is mid-week, and Performance if later in the week.

For step 4, data is carefully checked for variance. For example, in the lower right corner of Table 1, we note that the test performance of persons 4, 5, and 6 varies by way of scores 9, 8, and 7. However, if all scores were the same, the entire group would need to be removed prior to undertaking linear mixed-effects analysis. If the two-level structure in Table 1 were a dataframe in R (e.g., df), the following code would enable one to check for group variance: print(sort(tapply(df$Perform, df$Group, FUN = function(x)sd(x, na.rm = T))))

For step 5, the null and linear mixed-effects models are run using the statistical software of choice.

To validate our method, we have simulated some data in the R code [6] in Appendix A (Supplementary Material). The code also guides readers through the steps to complete the null and mixed-effects models. We run the null models (both two- and three-level) with the assistance of the R lme4 package [7], and optimization algorithms [8], [9] while the multilevel path models can be run with the R lavaan package [10].

## Declaration of Competing Interest

The authors declare that they have no conflicts of interest.

## Appendix Supplementary materials

Image, application 1
