# Supplementary material for: A protocol for analyzing repeated measures of online group behavior
Source: MethodsX. 2022 Mar 22;9:101667. doi: 10.1016/j.mex.2022.101667 (PMC8987404; doi:10.1016/j.mex.2022.101667)
Supplement: Supplementary file 1 [file mmc1.docx]

**Supplementary material:**

#################### Simulate Three-Level Data Structure #######################

install.packages("multilevel")

library("multilevel")

rm(list=ls())

set.seed(1234)

# Simulate 1000 person measures with five measures each per week, with variables (Volume & Performance) exhibiting ICCs 0.33, and a correlation between the 2 variables = 0.4

df <-multilevel::sim.icc(5,1000,0.33,2, item.cor = 0.4)

week <- rep(1:5, 1000) # Create associated week variable

group <- rep(c(1:40), each = 25) # Generate a 'group' variable for persons in this instance 40 groups with 125 observations

df <- cbind.data.frame(df, week, group) # Bind data

colnames(df) <- c("Person", "Volume", "Performance", "Week", "Group") # Name columns

df <- df[,c(4,1,5,2,3)] # re-order data: 5 observations per person, 25 persons in each of the 40 groups

####################### Run Three-Level Null Model #############################

install.packages("lme4")

install.packages("optimx")

library("lme4")

library("optimx")

NULL.MODEL <- lme4::lmer(Volume ~ 1 + # No predictors, only group mean fixed

(1 | Group/Person), # IDs nested inside groups with

data = df, # Defines the dataframe

REML= F, # ‘False’ optimizes for the log-likelihood

control = lmerControl(optimizer = "optimx", # For efficiency

calc.derivs = FALSE, # For speed

optCtrl = list(method = "nlminb") # For speed

) # END control function

) # END lmer function

summary(NULL.MODEL) # Prints summary

Var.comp <- print(VarCorr(NULL.MODEL), comp="Variance") # Extracts variance components from null model

Var.comp <- as.data.frame(Var.comp) # transforms to manageable dataframe

Person.var <- Var.comp$vcov[1] # Extract person-level variance

Group.var <- Var.comp$vcov[2] # Extracts group-level variance

Residual <- Var.comp$vcov[3] # Extracts residual (within person, weekly) variance

print(Group.var)

print(Person.var)

print(Residual)

total.var <- Group.var + Person.var + Residual # Sum total variance for model

Level3.ICC <- Group.var/total.var # Identify group intraclass correlation

Level2.ICC <- Person.var/total.var # Identify person intraclass correlation

round(Level3.ICC, 2) # 0.01 is the level 3 ICC

round(Level2.ICC, 2) # 0.32 is the level 2 ICC

round(1 - (Level3.ICC + Level2.ICC), 2) # 0.68 is the proportion of variance in Volume attributable to within person weekly variation

table(df$Group) # 40 groups, with 125 measures each

table(df$Person) # 1000 persons with 5 measures each

table(df$Week) # 5 weeks

################## Run Two-Level Null Model on Week 1 Data #####################

install.packages("lavaan")

library("lavaan")

rm(list=ls())

set.seed(1234)

df <-multilevel::sim.icc(25,40,0.33,3, item.cor = 0.4) # For Week 1, simulate 200 person measures in 5 groups, with variables (Volume & performance) to variables exhibiting ICCs 0.33, and a correlation between the 2 variables = 0.4

colnames(df) <- c("Group", "Volume", "Completeness", "Performance") # Name columns

NULL.MODEL <- lme4::lmer(Volume ~ 1

+ (1 | Group),

data = df,

REML=F,

control = lmerControl(optimizer = "optimx",

calc.derivs = FALSE,

optCtrl = list(method = "nlminb")

)

)

summary(NULL.MODEL)

Var.comp <- print(VarCorr(NULL.MODEL),comp="Variance")

Var.comp <- as.data.frame(Var.comp)

Group.var <- Var.comp$vcov[1]

Person.var <- Var.comp$vcov[2]

total.var <- Group.var + Person.var

Level2.ICC <- Group.var/total.var

round(print(Level2.ICC),2) # 0.39 is the ICC for Volume for Week 1

################## Run Two-Level Null Model on Week 1 Data #####################

grp.mean.vol <- tapply(df$Volume, df$Group, FUN=function(x)mean(x)) # Find group means for each group

grp.mean.freq <- unname(table(df$Group)) # Identify number of persons for each respective group

Volume.G <- rep(grp.mean.vol, grp.mean.freq) # Replicate means accordingly

grp.mean.per <- tapply(df$Performance, df$Group, FUN=function(x)mean(x)) # Find group means for each group

Performance.G <- rep(grp.mean.per, grp.mean.freq) # Replicate means accordingly

completeness.tot <- tapply(df$Completeness, df$Group, FUN=function(x)mean(x)) # Find group means for each group

Completeness.G <- rep(completeness.tot, grp.mean.freq) # Replicate means accordingly

df <- cbind.data.frame(df, Volume.G, Performance.G) # Bind new variables in dataframe

model <- '

level: 1

Performance ~ Volume

level: 2

Completeness.G ~ Volume.G

Performance.G ~ Completeness.G'

fit <- sem(model, df, cluster = "Group", std.lv=TRUE, estimator = "ML") # Run model

estim.M1 <- parameterestimates(fit, standardized=TRUE, rsquare=TRUE) # Extract standardized coefficients and R-squared estimates

print(estim.M1)

# END
